# Phenomenal Consciousness and Emergence: Eliminating the Explanatory Gap

**DOI:** 10.3389/fpsyg.2020.01041

**Published:** 2020-06-12

**Authors:** Todd E. Feinberg, Jon Mallatt

**Affiliations:** ^1^Icahn School of Medicine at Mount Sinai, Psychiatry and Neurology, New York, NY, United States; ^2^The University of Washington, WWAMI Medical Education Program, The University of Idaho, Moscow, ID, United States

**Keywords:** animal consciousness, explanatory gap, evolution, complex systems, physicalism, neurobiology, weak emergence, multiple realizability

## Abstract

The role of emergence in the creation of consciousness has been debated for over a century, but it remains unresolved. In particular there is controversy over the claim that a “strong” or radical form of emergence is required to explain phenomenal consciousness. In this paper we use some ideas of complex system theory to trace the emergent features of life and then of complex brains through three progressive stages or levels: *Level 1 (life), Level 2 (nervous systems)*, and *Level 3 (special neurobiological features)*, each representing increasing biological and neurobiological complexity and ultimately leading to the emergence of phenomenal consciousness, all in physical systems. Along the way we show that consciousness fits the criteria of an emergent property—albeit one with extreme complexity. The formulation *Life + Special neurobiological features → Phenomenal consciousness* expresses these relationships. Then we consider the implications of our findings for some of the philosophical conundrums entailed by the apparent “explanatory gap” between the brain and phenomenal consciousness. We conclude that consciousness stems from the personal life of an organism with the addition of a complex nervous system that is ideally suited to maximize emergent neurobiological features and that it is an example of standard (“weak”) emergence without a scientific explanatory gap. An “experiential” or epistemic gap remains, although this is ontologically untroubling.

## Introduction

Despite some of life’s unique features (Mayr, 2004) all basic life processes remain in principle explainable within the constraints of normal physics and chemistry. However, while the scientific basis of life is no longer a philosophical or scientific mystery, in the case of consciousness—more specifically in the case of subjective experience *(phenomenal consciousness, primary consciousness, raw “feelings” or irreducible “qualia”)* – there appears to be what philosopher Levine (1983) called an “explanatory gap” between the subjective experiences and the physical brain:

However, there is more to our concept of pain than its causal role, there is its qualitative character, how it feels; and what is left unexplained by the discovery of C-fiber firing is *why pain should feel the way it does*! For there appears to be nothing about C-fiber firing which makes it naturally “fit” the phenomenal properties of pain, any more than it would fit some other set of phenomenal properties. The identification of the qualitative side of pain with C-fiber firing (or some property of C-fiber firing) leaves the connection between it and what we identify it with completely mysterious. One might say, it makes the way pain feels into merely brute fact (Levine, 1983, p. 357).

In this paper, we discuss the critical role *emergence* plays in creating phenomenal consciousness and how this role helps explain what *appears* to be a scientific explanatory gap between the subjective experience and the brain, but which is actually not a scientific gap at all.

Note that we only consider basic, phenomenal consciousness (having any experience at all), not any higher types like reflective consciousness, self-consciousness, or higher-order cognition (Nagel, 1974, 1986; Block, 1995; Chalmers, 1995, 1996; Metzinger, 2003; Revonsuo, 2010; Churchland, 2013; Carruthers, 2016).

## What Is Emergence?

### General Features

Among the aforementioned features of life that Mayr (1982, 2004) discussed, the feature of emergence stands out as especially important for analyzing the creation of consciousness and the explanatory gap within a scientific framework. Emergence occurs when novel entities and functions appear in a system through self-organization. Our focus is on emergence in evolving complex systems as revealed by systems theory (Salthe, 1985; Morowitz, 2002; Ellis, 2006). We especially cover biology and neurobiology, although emergence can also apply to physical systems, mathematical and informational systems, philosophy, developmental psychology, and many other disciplines. For example, see the center manifold theorum of Carr (1981), the synergetics field of Haken (1983) and Tschacher and Haken (2007), the philosophical treatnents of Bedau and Humphreys (2008), the human-development focus of Beckermann et al. (2011) and Witherington (2011), and the general treatments by Simon (1973), Clayton (2006) and Clayton and Davies (2006).

**FIGURE 1:**
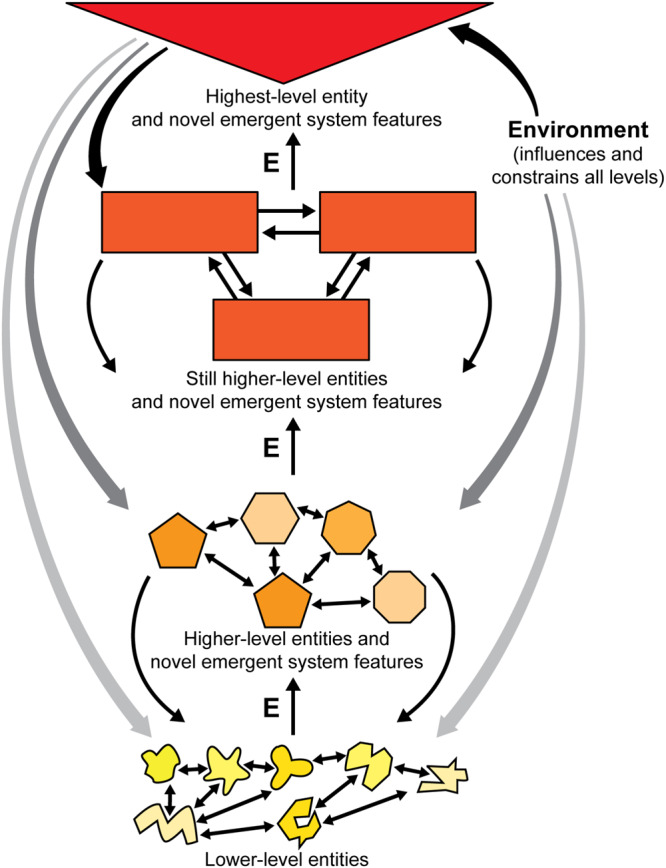
Emergence through a hierarchy in a complex system. Lower levels combine to make the higher levels. New features emerge (E) in the system as more levels are added. The many connections are reciprocal, as shown by the back-and-forth arrows, both between and within levels. Also see Table 1. Figure © Mount Sinai School of Medicine.

Modern formulations of emergence stem from efforts to understand the nature of *life* in the early part of the twentieth century, when it was realized that both the then-dominant hypotheses were scientifically inadequate: namely, vitalism (a mysterious life force) and reductionism (life can be explained mechanically as the mere sum of its parts) (Davies, 2006). With the concept of emergence, scientists could relinquish the idea of vital forces and also deny that life properties can be fully reduced to the mechanics of their parts. Instead they embraced a layered picture of nature consisting of ascending and interacting levels of increasing organizational complexity (Figure 1), with each higher level depending in part upon, but inexplicable in terms of, the properties of lower levels alone [adapted from Witherington (2011), after Broad (1925)]. Emergentism gained traction later in the century when complexity theory and detailed computer simulations generated many emergent features (Clayton and Davies, 2006).

Here in this “What Is Emergence?” section we summarize the general features of emergence in complex systems (Figure 1). Then in the next section, “A Model for the Emergence of Consciousness,” we will analyze how these general features contribute to the emergence of consciousness.

The following six features are often recognized as present in all emergent phenomena (Table 1).

**TABLE 1:** Major features of emergence in general.

1. Emergence is a property of *complex systems*, with many interacting parts
a. The interactions are *processes*, so processes are important (not just the physical parts)
2. *Aggregate system functions* that are not present in the parts alone
a. Whole is more than the simple sum of the parts; is not reducible to its individual parts
3. *Hierarchical arrangement* of different levels
a. Novel properties emerge in the system as higher levels are added
b. Emergent properties are novel properties
c. More novelty emerges if the system elaborates or evolves further
d. If the hierarchical system elaborates, there is more *specialization* of its parts and levels, both structurally and functionally
4. *Reciprocal connections* exist among structures within and between levels of the neural hierarchy
a. *Circular causality*: Lower levels bring about the higher levels, which then influence the lower levels (Salthe, 1985: Rothschild, 2006; Bedau, 2008; Nunez, 2016; Koch, 2019); and structures within the same level also influence each other via extensive reciprocal connectivity
5. *Constraints*:
a. The whole—and the emergent features of the system—constrain what the parts can do or be, and vice versa
b. External environment also constrains the whole and parts
c. Increasing a system’s complexity (more emergence) involves pruning the possibilities (Morowitz, 2002) to only those that let the system persist
6. There are *multiple routes to an emergent end-phenomenon*, from different sets of lower-level features (Bedau, 2008, pp. 181–182; Koch, 2019, pp. 122–124)

#### Features 1 and 2

Emergence occurs in *complex systems* in which novel properties emerge through the *aggregate functions* of the parts of that system. In the less complex of the complex systems, subatomic particles aggregate into atoms, which form molecules, etc. from which emerge all the nonliving chemical and geological processes. Favorite examples of such systems are the gravitational interactions among the heavenly bodies of the solar system, the turbulent flow of water, and weather systems (Morowitz, 2002; Nunez, 2016). Although we focus on complex *living* systems, these “simpler” systems fit the criteria for emergence and should not be forgotten.

A theoretical consequence for aggregate system functions is that the novel emergent functions cannot be explained by the parts *alone*, but rather must be explained by the properties of the parts *and* their interactions (Pattee, 1970; Allen and Starr, 1982; Salthe, 1985; Ahl et al., 1996; Bedau, 1997, 2008; Mayr, 2004; Clayton and Davies, 2006; Bedau and Humphreys, 2008; Beckermann et al., 2011).

#### Features 3 and 4

*Hierarchical arrangements* are particularly important in emergent systems because they allow *reciprocal connections* between levels where each higher or additional level gives the system novel emergent properties that are based on that level’s unique features as well as its interactions with the pre-existing (lower) levels on which it is built (Pattee, 1970; Allen and Starr, 1982; Mayr, 1982; Salthe, 1985; Ahl et al., 1996). For example, for our body to stay alive (highest level), our heart, its pumping muscle cells, and the energy-producing mitochondria in these cells (lower levels) must all interact reciprocally for the blood to be pumped.

This feedback entails *circular causality* between the levels of the system (Nunez, 2016; also see Haken, 1983). That is, emergence not only involves *bottom-up causation* by which the parts at the lower levels interact to cause novel (emergent) features at the higher levels, but it also involves *top-down causation* wherein the higher levels influence (constrain) the lower levels by making the lower levels subserve the whole system. For example, in a multicellular animal or plant, the organ and tissue components cannot act in ways that cause the organism to disassemble into its cells.

Circular causality is nicely incorporated in the Contextual Emergence Theory (Atmanspacher and beim Graben, 2009; Atmanspacher, 2012, 2015) and the Biological Relativity Theory (Noble et al., 2019). Both these theories emphasize top-down more than bottom-up causation, which is helpful for balance because it corrects past overemphases on the bottom-up causes in emergence (Witherington, 2011). Contextual emergence theory (CE), which is a scheme for describing a system’s relationships by comparing its higher and lower levels, offers additional insights. For example, CE shows that reductionist physicalism fails to explain nonlinear physical systems because their higher-level conditions (the “contingent context”) influence or stabilize or constrain the system’s lower-level mechanics, so the latter *alone* cannot explain the emergent properties (Bishop and Atmanspacher, 2006; beim Graben, 2014).

#### Feature 5

Emergence goes hand in hand with *constraint.* The system requirements themselves constrain what the parts can do: a living body cannot survive, for example, if some of its cells deprive others of vital resources (e.g. as occurs with a cancer) just as the external environment (e.g. extreme heat, cold, and aridity etc.) imposes constraints upon anything living under such conditions. And increasing a system’s complexity (meaning new levels and features emerge) involves *pruning the possibilities* to only those that let the system persist (Morowitz, 2002). As an example of this pruning, animals move and they evolved fast, Na+-based action potentials that signal neuromuscular-based mobility, whereas land plants are sessile autotrophs with rigid cell walls that prevent anything like neuronal branching or the extensive cell-to-cell communication of neural networks (Taiz et al., 2020). Therefore, even though land plants have evolved into enormously complex organisms they cannot use neuromuscular signaling like animals can. Stated in our terms, that option has been “pruned from” the plant lineage.

#### Feature 6

Finally, an end phenomenon may emerge through *multiple, alternate routes*. Two examples of this are traffic jams that can stem either from road construction or bad weather or a glut of vehicles (Bedau, 2008); and water waves that can stem from wind or an earthquake or a rock thrown into the water.

Bedau (2008, p. 181) called this alternate-routes feature “macro explanatory autonomy,” and it is akin to the psychological concept of *multiple realizability* (which says a given mind state can have different causes: Bickle, 2019). It also matches the biological concept of *convergent evolution* of similar traits in different clades of organisms (Stayton, 2015; Natarajan et al., 2016).

Another argument that multiple routes/multiple realizability is a feature of all complex systems comes from the above-mentioned contextual emergence theory. The argument is that the component parts of one system (“individual states, L_i_”) are allowed to differ in some ways from those in a second system that has the same emergent function (same “ensemble property,” such as consciousness), as long as the differing parts also share the *key similarities* that contribute to the emergent function (i.e. when the two sets of parts “are indistinguishable with respect to a particular ensemble property:” Atmanspacher, 2015: p. 360).

### Weak Versus Strong Emergence and Consciousness

The view that consciousness is an emergent process is not new (Lewes, 1877; Broad, 1925; Feigl, 1958; Popper and Eccles, 1977; Sperry, 1990; Searle, 1992; Scott, 1995; Bedau, 1997, 2008; Kim, 1998, 2006; Andersen et al., 2000; Feinberg, 2001, 2012; Van Gulick, 2001; Chalmers, 2006; Clayton and Davies, 2006; Thompson, 2007; Bedau and Humphreys, 2008; Beckermann et al., 2011; Deacon, 2011; Nunez, 2016; Mallatt and Feinberg, 2017). The important question for the nature of consciousness is: *what sort of emergence are we talking about? And what are its implications for the explanatory gap?*

While opinions vary on the relationship between emergence and consciousness, there are two main opposing schools of thought. One says that the operations of standard, scientific emergence that we have outlined (Table 1) can fully explain the emergence of consciousness. This is often described as the “weak emergence” theory (Bedau, 1997, 2008) or what Searle called emergence1 (Searle, 1992; Feinberg, 2001, 2012; Feinberg and Mallatt, 2016a). In this view, consciousness is or will be in the future fully understandable as an emergent property of micro-level brain process and the causal relations between them.

The other position is called strong emergence (Bedau, 1997, 2008; Chalmers, 2006; Clayton, 2006; Revonsuo, 2010) or emergence2 (Searle, 1992) or radical emergence (Feinberg, 2001; Van Gulick, 2001). It claims that no known properties of neurons could ever scientifically reconcile the differences between subjective experience and the brain; i.e. that the explanatory gap can never be closed. Antti Revonsuo nicely summarizes this position:

Supporters of strong emergent materialism point to the fundamental differences between the subjective psychological reality and the objective physical (or neural) reality. The former includes qualitative experiences that feel like something and exist only from the first-person point of view; the latter consists of physical entities and causal mechanisms that involve nothing subjective or qualitative about them and exist from the third-person point of view or objectively. Nothing we can think about or imagine could make an objective physical process turn into or “secrete” subjective, qualitative “feels.” It is like trying to squeeze wine out of pure water: it is just not there, and there can be no natural mechanism (short of magic) that could ever turn the former into the latter (Revonsuo, 2010, p. 30).

Next, we will explore the central role that emergence plays in the creation of consciousness. We then derive a “weak” or standard model and argue that the emergence of consciousness is simply a matter of the *degree* of standard emergence, not a different *kind* of emergence. Finally we analyze how and why the role of emergence in the creation of consciousness contributes to the *appearance* of a scientific explanatory gap that does not exist, but also that there is an *experiential* distinction or “gap” between first-person and third-person points of view.

## A Model for the Emergence of Consciousness

**FIGURE 2:**
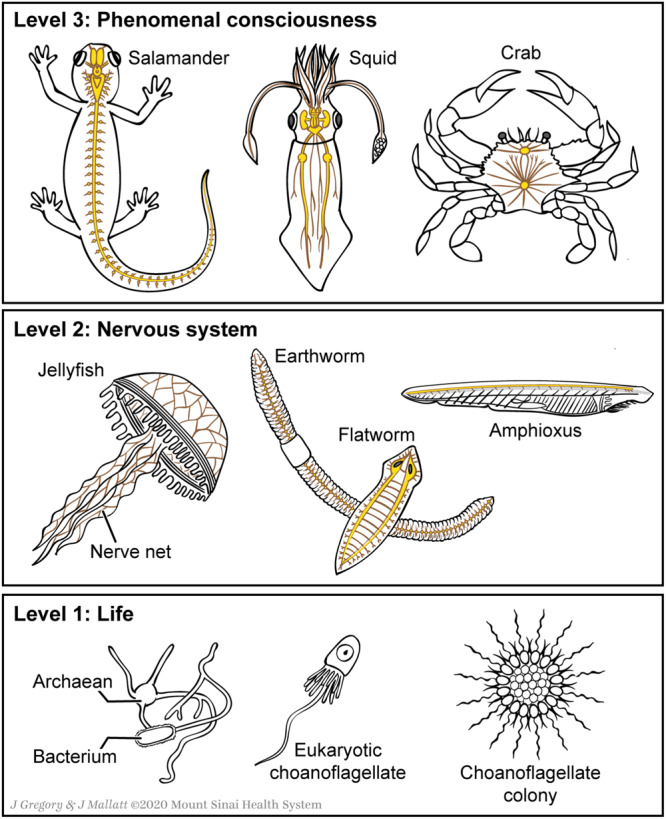
Organisms at the three emergent levels in the evolution of consciousness. Below, the colony of one-celled choanoflagellates shows how multicellular animals may have originated. Figure © Mount Sinai School of Medicine.

**FIGURE 3:**
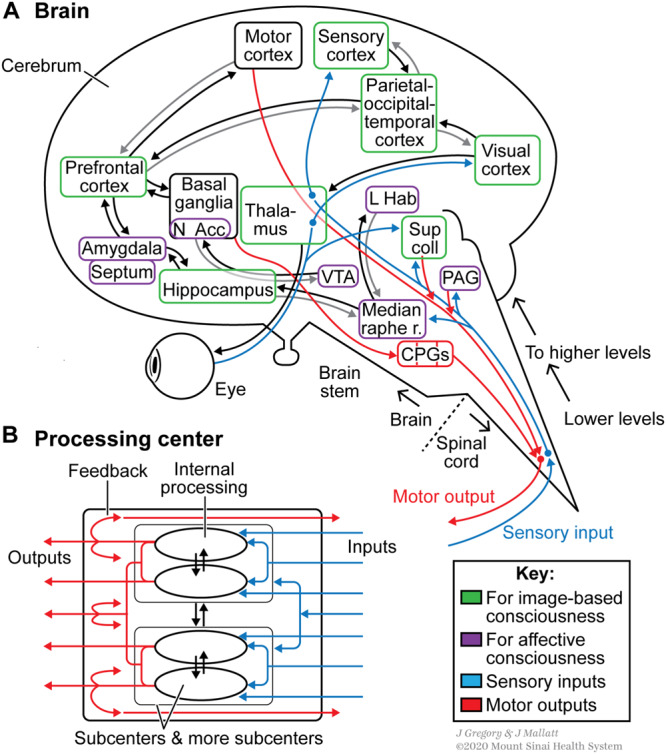
Some special neurobiological features of conscious systems, shown by the human brain and nervous system. These features include elaborate sensory organs (e.g. eye), neural hierarchical levels from the spinal cord upward, extensive reciprocal communication between neural processing centers (the rectangular boxes and the connecting arrows), and processing centers for image-based versus affective consciousness (green versus purple boxes). For more, see Table 2, **Level 3**. **(A)** Consciousness relies on processing centers that are widely distributed but integrated. While neural processing goes on *within* the centers, communication also occurs *among* the centers, leading to both local functional specialization and global coherence. **(B)** Schematic drawing showing processing within a center. The center has subcenters for subprocessing operations that are subsequently integrated to produce the center’s outputs. Abbreviations in **(A)** are CPGs: central pattern generators for various stereotyped movements; L Hab: lateral habenula; Median raphe r.: median raphe region of the reticular formation; N Acc: nucleus accumbens; PAG: periaqueductal gray; Sup coll: superior colliculus (optic tectum) of midbrain; VTA: ventral tegmental area of the midbrain. Figure © Mount Sinai School of Medicine.

Our model for the natural emergence of consciousness (Feinberg and Mallatt, 2013, 2016a,b, 2018a, 2019) has three levels. These are *Level 1 (life), Level 2 (nervous systems)*, and *Level 3 (special neurobiological features of consciousness)* that evolved in sequence and represent increasing biological and neurobiological complexity (Table 2). Each level displays novel emergent features, plus the features that emerged in the levels below it, plus the general features of all complex systems. Figure 2 shows some organisms at each level in the progression to consciousness, and Figure 3 shows some of the special features of consciousness at Level 3. We will now cover Table 2 step by step.

**TABLE 2:** Three emergent levels in the evolution of consciousness, and the new features at each level (adapted from Feinberg and Mallatt, 2019).

**Level 1. Life**
A. Simplest system that has life is the cell, with bacteria and archaea being the simplest cells
B. First appearance: ∼3.7 billion years ago
C. Emergent structures: macromolecules (proteins, nucleic acids, sugars, lipids), organelles, cells
D. Emergent processes:
∙ The strong boundary condition of *embodiment*: semipermeable membrane encloses cell contents to concentrate the chemical reactions and keep the reaction products from diffusing away (Morowitz, 2002)
∙ Information-based organization, directed by DNA/genes, and coded to specify the chemical reactions; the gene-coded “purpose” of Mayr (2004)
∙ Metabolism, to convert food to energy (ATP) and make new cellular materials; efficient use of energy and of vital molecules slows entropy (energy waste lost as heat)
∙ Self-upkeep and goal-directed properties (Mayr, 2004; Godfrey-Smith, 2019)
∙ Growth and self-replication/reproduction
∙ Sensitivity and movement
∙ Homeostasis: maintaining a constant internal environment in response to changes in the external environment
∙ Adaptation to the environment
∙ Evolution; natural selection becomes the pruning process that limits the possibilites of evolutionary change and of what features emerge in the system from this level onward (Morowitz, 2002)
E. Adaptive advantage of this emergence: world’s first self-perpetuation of complex systems over time
**Level 2. Nervous systems, From Reflexes Through the Level of Simple, Core Brains (Not Conscious)**
A. Organisms possessing it: most invertebrate animals; for example, most worms
B. First appearance: ∼ 580 million years ago
C. Emergent structures: multicellular animal body with different cell types including neurons, neural reflex arcs, sensory receptors, motor effectors (muscles, glands); nerve nets, then a consolidation into central and peripheral nervous system; some of the animals have a simple brain with movement-patterning circuits; the sensory receptors are mechano-, chemo- and photoreceptor cells
D. Emergent processes:
∙ Speed: neurons transmit signals fast enough to control the actions of a large, multicellular body in response to sensory stimuli
∙ Connectivity: reflex arcs and neuron networks coordinate all the parts of a large body
∙ Core-brain processes:
∘ Control complex reflexes for inner-body homeostasis
∘ Basic motor programs and central pattern generators for rhythmic locomotion, feeding, and other stereotyped movements
∘ Set the level of arousal
E. Adaptive advantages of this emergence: Sustains a large body that can move far through the environment, following sensory stimuli to find food, safety, and mates
**Level 3. Consciousness**
A. Organisms possessing it: vertebrates, arthropods, cephalopod molluscs
B. First appearance: 560–520 million years ago
C and D. Emergent structures and processes: the *special neurobiological features of consciousness:*
∙ Neural complexity (more than exists in a simple, core brain)
∘ Brain with many neurons (>100,000?)
∘ Many subtypes of neurons
∙ Elaborated sensory organs
∘ Image-forming eyes, receptor organs for touch, hearing, smell
∙ Neural hierarchies with neuron-neuron interactions
∘ Extensive reciprocal communication in and between the pathways for the different senses
∘ Brain has many neural computing modules and networks that are distributed but integrated (separate but highly interconnected), leading to *local* functional specialization plus *global* coherence (Nunez, 2016; Mogensen and Overgaard, 2017) (see Figure 3)
∘ Synchronized communication by brain-wave oscillations; neural spike trains form representational codes
∘ The higher levels allow the complex processing and unity of consciousness
∘ Higher brain levels exert more influence on the lower levels such as motor neurons, for increased top-down causality
∘ Hierarchies that let consciousness model events a fraction of a second in advance (Clark, 2013; Gershman et al., 2015; Jylkkä and Railo, 2019; Solms, 2019)
∙ Pathways that create mapped mental images or affective states
∘ Neurons are arranged in topographic sensory maps of the outside world and body structures
∘ Valence coding of good and bad, for affective states
∘ Feed into premotor brain regions to motivate, choose, and guide movements in space
∙ Brain mechanisms for selective attention and arousal
∙ Memory, short-term or longer
E. Adaptive advantages of this emergence:
∙ Consciousness organizes large amounts of sensory information into a detailed, unified simulation of the world, so the subject can choose the best behavioral responses
∘ This is a large, effective, expansion of the basic life-property of sensing the environment and responding
∙ With mental maps, one can navigate through space even when no sensory stimuli for guidance are present
∙ Consciousness ranks all the sensed stimuli by importance, by assigning affects to them (good, bad), thereby simplifying decisions on how to respond (Cabanac, 1996)
∙ Consciousness provides behavioral flexibility: adjusts fast to new stimuli so it deals well with the changing challenges of new environments

### Level 1. General Life Functions

Living systems are replete with examples of emergent system features (Mayr, 1982, 2004; Salthe, 1985; Morowitz, 2002; Rothschild, 2006; Van Kranendonk et al., 2017). Even the simplest one-celled life involves chemical reactions far more complex than in any known nonliving system, and fossils indicate that life on Earth has been around for a long time – arising in seas, springs or ponds at least 3.7 billion years ago. From simple organic molecules must have arisen a boundary membrane, providing *embodiment* to form a protocell. This boundary enclosed and contained the molecules that used energy for vital processes (the catalytic and substrate molecules for *metabolism*) plus the *information molecules* RNA and DNA that instructed these processes and allowed the protocells to sustain and reproduce themselves (England, 2013). Only those protocells that sustained themselves long enough and reproduced often enough avoided the destructive vicissitudes of the external environment. This led to a competition for survival that favored those cells that most efficiently maintained their internal chemistry (*homeostasis* based on cooperating subcellular systems) and also were best *adapted* to the external environment. This was the first organic evolution by natural selection and it has driven life’s adaptations over billions of years, including the emergence of increased complexity in higher organisms. Natural selection also limited (constrained) the directions that living organisms could take, to those changes that are compatible with organic-based and water-based life.

From this, we reiterate that life itself is an emergent process created by the constituent parts of the organism. So for instance the life of a single cell is an aggregate emergent feature of the atoms, molecules, proteins, membranes, ribosomes, etc. of which it is composed and their interactions.

Evolution proceeded over billions of years in one-celled organisms. Then more complexity emerged in some marine cells about 1.5 billion years ago when one type (perhaps akin to today’s microbes called archaea) engulfed a species of bacterium that was especially efficient at extracting energy from nutrients, so those bacteria became the energy-producing mitochondria within a new, larger, symbiotic system called the eukaryotic cell. Some eukaryote cells joined into large, multicellular groups – likely because larger organisms are harder for predators to kill and eat—that evolved into the first animals 700 or 600 million years ago. These first animals may have resembled immotile sponges, but the ability to move followed because movement offered great advantages for reaching the best places in the environment for food, mates, and safety. All this led to selection for a specialization of cells within the multicellular body, for a division of labor, into muscle cells, gut-digestive cells, sex cells – *and nerve cells to coordinate the activities of the muscle and all the other cell types*. For accounts of this evolutionary sequence of emergent features, see Lane and Martin (2010), Feinberg and Mallatt (2016a), Brunet et al. (2019), Brunk and Martin (2019), and Watson (2019).

### Level 2. Nervous Systems, Reflexes, Core Brains

Judging from modern cnidarians (jellyfish and their kin: Figure 2) and some simple marine worms, the first nervous systems were nerve networks distributed over the body, without any central or brain-like structures. The neurons communicated quickly (nerve fibers carry their signals at 0.5 to 100 m per second) and tightly (with synapses), to produce fast reflexes and effective movements. Thus, the whole body participated in receiving sensory stimuli and in the resulting motor reactions. The animals at this stage had sensory mechanoreceptor cells for touch stimuli, basic chemoreceptor cells for tastes and scents, and photoreceptor cells for light intensity (but no visual images in this eye-less stage).

Then around 580 to 520 million years ago the worm ancestors gave rise to many groups of animals, including most of the invertebrate groups and the vertebrates. In many of these descendant lineages, parts of the nerve net condensed and enlarged for information processing, most so in the head region that received sensory information first as the animal moved forward through its environment; and from these neural enlargements there extended nerve cords that carried motor commands along the body axis. These were the first brain and nerve cord of an incipient central nervous system. Many living invertebrates reflect this incipient stage (e.g. roundworms, earthworms, flatworms, sea slugs, and the fish-like cousin of vertebrates called amphioxus: Figure 2). Such invertebrates have relatively simple “core brains” that integrate sensory information, adjust inner-body processes (digestion, sex activity of the gonads, hormone secretion), and set the animal’s overall level of arousal (placid, excitable). Core brains also contain basic motor programs for rhythmic locomotion, feeding movements, and other stereotyped actions. For accounts of the sequence of emerging neural features just described, see Feinberg and Mallatt (2016a), Bosch et al. (2017), Shigeno et al. (2017), Lacalli (2018), and Arendt et al. (2019).

Elaborate neural connections and many behaviors emerged at this core-brain stage, but it is not conscious. We deduce this because it is entirely *reactive* and therefore, reflexive. The invertebrates at this stage sense and follow stimuli that are essential to their survival, but if they lose the sensory trail — with no more stimuli to react to — they cannot go further and resort to systemic but untargeted searching to try to relocate the stimulus. See the evidence for this from foraging roundworms by Klein and Barron (2016) and Feinberg and Mallatt (2018a). Consciousness evolved to solve this problem of becoming lost, and it involved acquiring a new set of emergent features.

### Level 3. the Special Neurobiological Features of Consciousness

#### How We Deduced These Features

The *special neurobiological features* of complex brains, combined with the more basic life functions, reflexes, and core brain, create consciousness (Table 2, **Level 3**). Before putting these special features into an emergent evolutionary scenario, we should tell how we derived them. They are our versions of the “neural correlates of consciousness” or NCCs, namely our minimal set of neuronal traits that are collectively sufficient for consciousness (Edelman et al., 2005; Searle, 2007; Seth, 2009; Koch, 2019). NCCs are the traits that all investigators must establish before they can study consciousness any further. Whereas most other investigators base their correlates on studies of the mammalian or human cerebral cortex — as if consciousness only emerged with or in the cortex — we instead derived our correlates from two fundamental assumptions (Feinberg and Mallatt, 2013, 2016a, 2018a, 2019): (1) If an animal has neural pathways that carry mapped, point-by-point signals from the sensed environment, from different senses (e.g. vision, touch, hearing), and if these sensory maps converge in the brain, then that animal consciously experiences a unified, mapped, multisensory image of the environment; and (2) If an animal shows complex operant learning, i.e. learning and remembering from experience to avoid harmful stimuli and to approach helpful stimuli, then that animal has the negative and positive feelings of affective consciousness (also see Bronfman et al., 2016 and Ginsburg and Jablonka, 2019). The only animals that meet these two criteria are the vertebrates, arthropods, and cephalopod molluscs (octopus, squid, cuttlefish) (Figure 2). After recognizing this, we sought and tallied the other novel neural features shared by all three of these taxa, to complete our list of special features in Table 2, **Level 3**.

#### The Special Features Are Emergent Features

The special features of consciousness in Table 2, **Level 3** fit all the criteria for emergence in Table 1. Consciousness fits Features 1 and 3 of Table 1 because it is a novel *process* that comes from a *complex, hierarchical system* of living and nervous elements, with its novelty attained through addition of the special neural features; and it is *not present in the system’s parts* such as in an individual neuron nor the ancestral, core brain (Feature 2 in Table 1).

With consciousness, there is more elaboration, specialization and subdivision of the hierarchy’s parts (Feature 3d in Table 1). The first example of this is that the senses of vertebrates, arthropods and cephalopods are much more elaborate than the simple ancestral photorecepters, mechanoreceptors and chemoreceptors, in including image-forming eyes, ears for hearing, taste buds, and olfactory organs (Feinberg and Mallatt, 2016a). Second, the sensory pathways have more levels (levels added to the hierarchy), namely the brain’s higher-processing and motor-command centers. The best example of this is that the vertebrate brain has new levels in its highest part (forebrain) that were not present in prevertebrates as judged from the brains of our invertebrate cousins, the amphioxus and tunicates (sea squirts). More specifically, only the vertebrates have an enlarged and complex cerebrum in their forebrain.

As a third example of the great elaboration and specialization associated with consciousness, the more-advanced animal brains have the largest numbers of neuron *types*, with highly complex interactions (Strausfeld, 2012; Feinberg and Mallatt, 2016a; Hodge et al., 2019). As a fourth example, the brains of conscious animals have many more brain regions than do the ancestral core brains. Some of these added regions process the extensive sensory inputs. In vertebrates, for instance, visual information is extensively processed in the retina, thalamus, parts of the cerebrum, and optic tectum; and in arthropods in the retina, lamina, medulla, lobula and central complex of the brain (Strausfeld, 2012; Feinberg and Mallatt, 2016a). As another illustration of extreme regional specialization, the core of the vertebrate brain has elaborated a dizzying number of centers for affective (emotional) consciousness: the habenula, basal forebrain, periaqueductal gray, parts of the reticular formation and more (Figure 3; Berridge and Kringelbach, 2015; Feinberg and Mallatt, 2016a; Hu, 2016; Feinberg and Mallatt, 2018a; Siciliano et al., 2019; Szõnyi et al., 2019).

The special neural features of consciousness include the emergent feature of more *reciprocal connections* (Feature 4 in Table 1). For this, the functional centers communicate back and forth through extensive interconnections (Figure 3), commonly by synchronized oscillatory signals or reverberations (Lamme, 2006; Koch et al., 2016; Feinberg and Mallatt, 2018a; Koch, 2019). Lack of extensive cross-communication is thought to be why some of our complex brain regions operate *non*consciously; an example is the cerebellum, which nonconsciously smooths and coordinates our body movements (Tononi and Koch, 2015).

The related property of *circular causality* (Feature 4a) is more pronounced in conscious than in core-brain systems. For example, the lower levels that receive sensory input influence the higher brain levels that in turn dictate motor output, and they do so far more extensively than in the more reflex-dominated nervous systems of nonconscious animals (Grillner and El Manira, 2020).

Extensive reciprocal communication also allows consciousness to be an effective *prediction device*, modeling events a fraction of a second into the future so the subject is always prepared in advance. Stated briefly, all this crosstalk lets the hierarchy continuously sense the current events, make the predictions, and perpetually adjust these predictions to optimize the behaviors the hierarchy signals. Predictive processing is a large focus of consciousness research nowadays, and we explain more about it in our book, *Consciousness Demystified* (Feinberg and Mallatt, 2018a).

Consciousness and the neural features that support it come with *constraints* (Feature 5 in Table 1). The complex neural processes are energy expensive. Due to this cost constraint, (1) a conscious individual cannot attend to every stimulus that is sensed but must instead use *selective attention* (see for instance Tsuchiya and Koch, 2008; Chica et al., 2010; Block, 2012; Tsuchiya and Van Boxtel, 2013; Koch, 2019) that might miss some important stimuli; (2) some brain processes must run on automatic without consciousness, such as those for swallowing and well-practiced motor skills; (3) many bilaterian animals never evolved consciousness due to its cost, having instead evolved shortcuts for survival, defense and finding food (e.g. the tiny-brained, filter-feeding clams in their protective shells).

**FIGURE 4:**
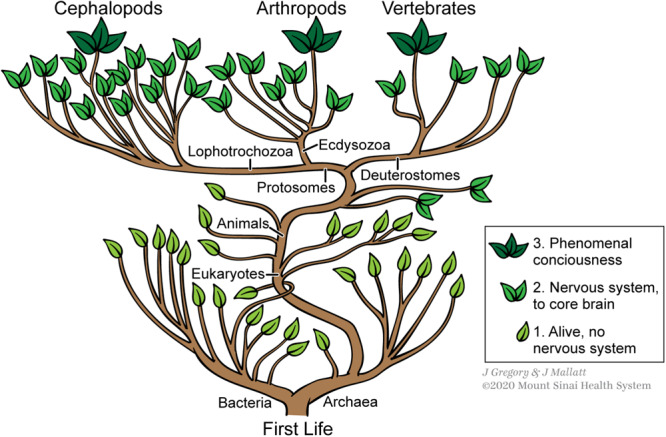
The phylogenetic “bush of life” showing that consciousness (as three-leaved stems) emerged independently in three different lines of animals. Figure © Mount Sinai School of Medicine.

As for the *multiple routes* feature of emergent phenomena (Feature 6 in Table 1), this is exactly what we found for consciousness (Feinberg and Mallatt, 2016a, b, 2018a), in the above-mentioned form called the *multiple realizability* of a mental state. For example, the complex brains of vertebrates, arthropods, and cephalopods – each of which has *all the special features* of consciousness – evolved independently of one another from a brainless ancestral state (Northcutt, 2012), meaning their consciousnesses evolved by three different routes (Figure 4). As another example of the multiple realizability of consciousness, in mammals the mapped, conscious images of the sensed world primarily involve a different part of the brain (cerebral cortex) than does the affective consciousness of emotions (subcortical brain regions) (Panksepp, 2004, 2016; Denton, 2005; Merker, 2007; Damasio, 2010; Aleman and Merker, 2014; Berridge and Kringelbach, 2015; Feinberg and Mallatt, 2016a).

Significantly, we have reported this “diversity” of the conscious substrates in the past (Feinberg and Mallatt, 2016a, b, 2018a, 2019), but we only recently recognized it as the multiple-routes and multiple-realizability feature, and therefore as a hallmark of emergence in complex systems in general (Bedau, 2008).

#### Dating When the Special Features Emerged

From the evidence that consciousness is confined to vertebrates, arthropods, and cephalopods, it is easy to deduce when consciousness emerged and to see that it did so rapidly, in evolutionary terms. The earliest arthropod, vertebrate and cephalopod fossils are from Cambrian rocks, products of the “Cambrian explosion” that produced all 30+ known phyla of bilaterian animals between about 540 and 500 million years ago (Erwin and Valentine, 2013). This explosion is thought to have been sparked by the evolution of the first predatory animals (the earlier, ancestral worms had fed on sea-floor scum), leading to an adaptive arms race that yielded many distinct taxa with different defensive and predatory strategies. Judging from their modern relatives with similar locomotory and sensory morphologies, the Cambrian arthropods, vertebrates, and cephalopods were highly active and far-ranging animals that could navigate through space to find food and mates, and avoid danger. By this reasoning, they all must have had the mapped mental images of the environment that signify conscious awareness. Consciousness was a big advance that also contributed to the *further* (later) success of these taxa: arthropods have always been an extremely diverse and abundant phylum, and vertebrates include the largest animals with the biggest brains, at the top of the food chain. For documentation of these ideas, see Plotnick et al. (2010), Trestman (2013), Feinberg and Mallatt (2016a), Godfrey-Smith (2016), Godfrey-Smith (2019), Klein and Barron (2016), and Ginsburg and Jablonka (2019).

To summarize, this section shows that consciousness evolved in some animals along with an elaboration of their body plans, and it did so in the Cambrian Period as a key adaption in the history of life on Earth. Many special neural features evolved with it, and these features fit the criteria of emergent features in general (Table 1). This close fit implies our special features of consciousness *really are* emergent features. Yet these features are highly elaborated extensions of those in simpler brains so they reflect huge increases in emergent novelties.

## Emergence, Consciousness, and the Explanatory Gap: Some Philosophical Implications

### The Case for the Weak (Natural) Emergence of Consciousness

We have argued, by comparing the general features of emergence (Table 1) with the features that appeared during the evolution of consciousness (Table 2), that consciousness is a naturally emergent feature of life and complex brains. We summarize this formulation as *Life + Special neurobiological features → Phenomenal consciousness*. The neural-reflexive stage (Level 2) serves as an evolutionary and neurobiological bridge between *Life* and the *Special features*.

However, as we noted above, the idea that consciousness is an emergent process is not new. The important question here is whether we are correct in concluding that consciousness is produced by “standard” emergent principles that are amenable to standard scientific investigation, and is thus an example of weak emergence. In other words, can we explain the emergence of consciousness in a seamless way *with no scientific explanatory gap* between life and consciousness?

We will now argue that the two main factors – life and the special features – make crucial contributions to the creation of consciousness but at the same time they contribute to the (mistaken) appearance of a scientific explanatory gap where none actually exists.

### Life Is Crucial to Explaining the Subjective Aspect of “Feeling”

First we consider the role of life. Note that in Levine’s (1983) view and that of many philosophers of consciousness (Broad, 1925; Nagel, 1974, 1986; Chalmers, 1995, 1996; Shear, 1999) it is the *subjective*, *personal* nature of consciousness that makes it so perplexing and mysterious and makes strong emergence seem like a reasonable – if not a default – position. So how can the personal subjective nature of consciousness be explained by objective neurobiological science?

First, because consciousness is built upon the emergence of life in any single organism, and because both life and consciousness are *system features of embodied organisms*, then it follows that conscious feelings (perceptions, “qualia,” etc.) are system functions of certain complex, personal brains, and each feeling is a personal system-feature of that individual living organism just as life itself is an embodied personal system-feature of the organism. Therefore, life provides the natural *initial conditions* for the emergence of subjective consciousness (Feinberg, 2012; Feinberg and Mallatt, 2016a, b, 2018a). In short, life means embodiment, which means an individual body, which ultimately allows an individual perspective (subjectivity).

But as Thompson (2007) notes, the explanatory gap by no means goes away simply because consciousness is a feature of life. Not all living organisms or body organs are conscious (see Mallatt and Feinberg, 2017), so life only partly fills that gap. To finish filling the gap, the special neurobiological features of conscious brains are needed to explain what is unique about consciousness. These features are personal but also novel.

### Some Brains Are Ideally and Uniquely Suited to Create Novel Emergent Features

Our main finding so far is the remarkable correspondence between the special features of conscious brains listed in Table 2, **Level 3** and the general features of emergence in *all* complex systems listed in Table 1. The special features not only correspond to the general features but markedly *extend* them, to levels of much greater complexity. This provides good evidence that consciousness is complex, with complex causes, and is not simply caused by one fundamental, or psychic, force of nature as some have claimed (Chalmers, 1996; Velmans, 2008; Skrbina, 2009; Goff et al., 2017).

Another way to say this is that when we compare Table 1 with Table 2, **Level 3**, we find that the neural hierarchies for consciousness are ideally suited to maximize emergent novelty.^*^ These neural hierarchies have large numbers of tightly and reciprocally connected neural levels and centers, which interact extensively, and enhanced neuron-neuron communications that maximize the distributed yet interconnected neural levels (Figure 3). They also have an enormously increased differentiation of neuron subtypes in the setting of enhanced aggregate functioning. This is much more elaborate than in less-complex systems because of its greater number of interacting parts.

Thus, while it has been proposed that consciousness requires a strong type of emergence that is different in *kind* from standard (weak) emergence, we see that the emergence of consciousness is simply a matter of a greater magnitude of standard emergence with an accompanying exponential increase in novel emergent properties. Such a large quantitative increase gives the impression of a *qualitative* explanatory gap between the brain and consciousness when there actually is none. This realization, along with the personal point of view that comes from embodied life, is our solution to the longstanding problem of the explanatory gap. But we will see later that a different gap remains, though that gap is fully explainable as well.

### Consciousness, Emergence and Downward Causation

Another assumption that contributed to the idea of an explanatory gap is the view that consciousness emerges “at the top” of the neural hierarchy. According to one version of this view, the “mental properties” that emerge at the highest level can then cause “physical changes” in a downward fashion upon the material brain. (For a discussion of this and other accounts of downward causation and consciousness, see Emmeche et al., 2000).

In a prototypical example of this kind of theory, Nobel laureate Sperry (1984, 1990) argued that the “mysterious” features of consciousness are radically/strongly emergent, non-material features of the brain:

… consciousness was conceived to be a dynamic emergent of brain activity, neither identical with, nor reducible to, the neural events of which it is mainly composed. Further, consciousness was not conceived as an epiphenomenon, inner aspect, or other passive correlate of brain processing, but rather to be an active integral part of the cerebral process itself, exerting potent causal effects in the interplay of cerebral operations. In a position of top command at the highest levels in the hierarchy of brain organization, the subjective properties were seen to exert control over the biophysical and chemical activities at subordinate levels. It was described initially as a brain model that puts “conscious mind back into the brain of objective science in a position of top command … a brain model in which conscious, mental, psychic forces are recognized to be the crowning achievement … of evolution (Sperry, 1990, p. 382).

And:

For the subjective qualities we look higher in the system at organizational properties that are select and special to operations at the top levels of the brain hierarchy (Sperry, 1984, p. 671).

Feinberg (2001) pointed out the error in this analysis. While consciousness is clearly an emergent feature of complex brains, it is a *system feature*, and as such does not emerge at the “top” *or any other “point” of the neural hierarchy*. It is a product of the entire system and many levels contribute.

The view of a strongly emergent – but immaterial – feature that somehow “pops out” at the summit of the nervous system contributes to the idea of an explanatory gap (see the Revonsuo, 2010, quote above) that in reality does not exist (Feinberg, 2001). It also contributes to the mistaken, dualistic, claim that immaterial consciousness miraculously controls the material brain.

### Consciousness, Multiple Realizability, and Emergence

We have provided evidence for the *multiple realizability* of consciousness, which is the idea that a given mental state can have different causes. This concept was put forth by Putnam (1967). He introduced it as a rebuttal of the strictly reductionist idea that the mental is identical to the physical. Such a reductionist identity could allow just *one* physical cause for a mental state, not multiple causes, so multiple realizability was an effective rebuttal. Its appeal led many physicalist philosophers to become *nonreductive physicalists* (Kim, 1992; Bickle, 2019).

However, extensive analyses of the multiple-realizability concept over the decades have led some scholars to question its premise that a multiply realizable mental state is a single entity or “kind” (Block, 1980; Kim, 1992; Endicott, 1993). For example, pain in insects does not have the same physical basis as pain in humans, so these two types of pain could actually be called two distinct entities. This challenge would mean that *every* multiply realizable mental state is a composite or conjunction of states (if from different species), and in being a mixture is not amenable to scientific analysis—which Kim (1992) claimed prevents psychology from being a scientific discipline.

Our findings refute this challenge for the particular mental state of consciousness. We found that every multiply realizable conscious system—in vertebrates versus arthropods versus cephalopods, and for affective- versus image-based consciousness—has a large number of physical features *in common*, all of which are listed in Table 2, **Level 3**. The commonalities are so numerous that consciousness, we argue, despite its variations, can indeed be treated as a single mental kind. Another criterion for a mental state to be a single kind is if all its variations have the same causal powers (Kim, 1992), and we demonstrated this too, in that the conscious state causes active, directed behaviors in all the conscious taxa (see above). The many, unifying regularities we uncovered for the conscious state are not coincidental or trivial, but instead comprise a suite of essential adaptations, convergently molded by the selective evolutionary constraints needed for highly mobile animals to operate proactively in a directed manner in complex environments.

These considerations demonstrate the value of multiple realizability in consciousness studies and psychology in general—and we have shown that multiple realizability comes directly from the *multiple-routes feature* of all emergent systems (Table 1, Feature 6).

### Consciousness, Emergence, and the “Experiential Gap”: Being Versus Describing

While we find no scientific explanatory gap between the brain and subjective experience from the standpoint of biology and neurobiology, we acknowledge that there remains an *experiential (“point of view”) gap* between objective scientific explanations of the brain and subjective experiences of consciousness. The question is whether this gap causes a problem for a complete *science* of consciousness.

To illustrate the experiential gap, C. D. Broad argued that even if an omniscient “mathematical archangel” could fully explain the chemistry of ammonia and the functions of the brain, the archangel still could not predict the subjective *smell* of ammonia:

He [the archangel] would know exactly what the microscopic structure of ammonia must be; but he would be totally unable to predict that a substance with this structure must smell as ammonia does when it gets into the human nose. The utmost that he could predict on this subject would be that certain changes would take place in the mucous membrane, the olfactory nerves and so on. But he could not possibly know that these changes would be accompanied by the appearance of a smell in general or of the peculiar smell of ammonia in particular, unless someone told him so or he had smelled it for himself (Broad, 1925, p. 71).

We fully agree with Broad that no amount of *explanation* of the neurobiology of the brain can *eliminate* the need for the subjective aspect of personal experience, any more than *describing* one’s first-person experience can substitute for *having* that experience. And we agree that no amount of indirect *knowledge* or *description* of brain functions can be equated with, fully capture or can substitute for “*something it is like to be,” phenomenal consciousness*, the *first-person versus third-person point of view*, or *knowledge by acquaintance versus knowledge by description* (Russell, 1910, 1912, 1914; Nagel, 1974, 1986; Jackson, 1982, 1986; Levine, 1983; Velmans, 1991; Searle, 1992, 2007; Conee, 1994; Block, 1995; Chalmers, 1995, 1996; Metzinger, 1995, Metzinger, 2003; Tye, 2002; Revonsuo, 2006; Teller, 2011; Carruthers, 2016; Choifer, 2018; Hasan and Fumerton, 2019; Nida-Rümelin and O Conaill, 2019).

So how do we scientifically reconcile the experiential divide between these first- and third- person points of view without invoking any *dualism* between the brain and the mind? How can the divide be compatible with physicalism? (See for instance the “knowledge argument” against physicalism: Jackson, 1982, 1986; Conee, 1994; Nida-Rümelin and O Conaill, 2019).

**FIGURE 5:**
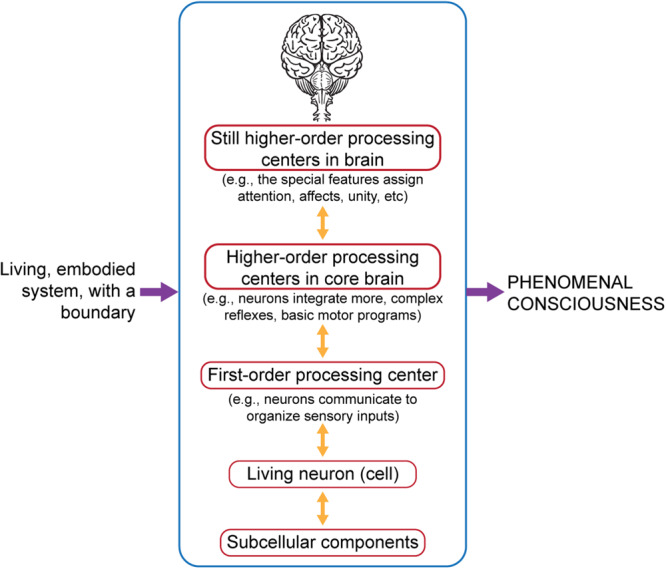
Phenomenal consciousness is an emergent system function that relies on neural hierarchies and also on embodied life (Table 2, **Level 1**) and special neurobiological features (Table 2, **Level 3**). Our formulation summarizes this: Life + Special neurobiological features → Phenomenal consciousness. Figure © Mount Sinai School of Medicine.

Here is our answer. If it is true, as we propose, that the *personal life* of an embodied organism is an emergent process of a physical system (Table 1 and Table 2, **Level 1**), then subjectivity is a critical but *biologically natural* element of what we experience as a phenomenal state; and if it is also true, as we propose, that the addition of the special neurobiological features of complex brains (Table 2, **Level 3**) provides the *biologically natural* elements necessary for the hierarchical emergence of phenomenal consciousness, then we have enumerated all the prerequisites that are required for the natural emergence of subjective experience (Figure 5).

**FIGURE 6:**
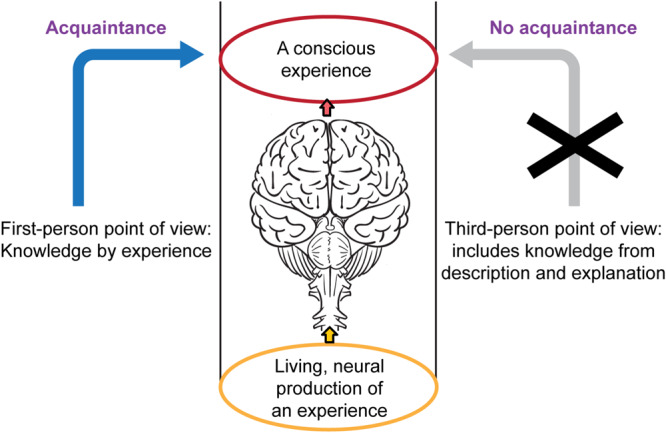
Some kinds of knowledge can only be obtained by experience. Knowing is of two types, experiential (left) and descriptive (right). An observer cannot fully know an experience (X at the right) without directly experiencing it, even though the experience is generated physically by neurons in a living brain (center column). The distinction here between first- and third- person points of view does not entail dualism between the brain and the mind or require a “non-physical” explanation for phenomenal consciousness. Also see Feinberg and Mallatt (2018b). Figure © Mount Sinai School of Medicine.

Thus we find that the distinction between *being and experiencing* versus *observing and describing* is accounted for by phenomenal consciousness as an emergent feature of living complex brains (Figure 6). This means the “knowledge by description” of phenomenal consciousness – as sought by Broad’s archangel—is different from direct “*knowledge by acquaintance”* or *phenomenal knowledge* because some kinds of knowledge can only be obtained through experience, even in a completely physical world (Conee, 1994). By this account, the “experiential gap” does not violate physicalism, nor does it support the strong emergence of consciousness.

## Conclusion

In summary, our proposed solution to the explanatory gap is that first, the emergence of phenomenal consciousness has a scientific explanation that adheres to and is consistent with the principles of emergence in the rest of nature. A close consideration of the special features of conscious systems (Table 2, **Level 3**) shows these features fit all the criteria of emergent features of complex systems in general (Table 1), thereby confirming that consciousness is a complex-systems phenomenon, and that it is not just one thing arising from one cause, such as a new “fundamental” physical force of nature (Chalmers, 1995, 1996).

Second, our formulation for consciousness as a physically emergent process is *Life + Special neurobiological features → Phenomenal consciousness*, in which the (personal) *Life* aspect is the ultimate basis of subjectivity and the *Special features* aspect is the necessary additional basis of conscious experiences. We show how this formulation explains consciousness as an instance of standard, weak emergence without a need for strong emergence or a scientifically unbridgeable explanatory gap.

Third, with the natural emergence of consciousness thus explained, the only remaining gap is a mere “experiential gap” between first-person experience and third-person description that poses no obstacle for a naturalistic explanation of consciousness.

## Author Contributions

TF focused more on the neurobiology, theory and philosophy. JM focused more on the neurobiological and evolutionary aspects. Both authors contributed to the emergent features and their relation to consciousness.

## Conflict of Interest

The authors declare that the research was conducted in the absence of any commercial or financial relationships that could be construed as a potential conflict of interest.

## Footnotes

* See Jordan and Ghin (2006: p. 64) for another perspective on why the novel emergent features for consciousness can accumulate and then accelerate over evolutionary time in living, self-sustaining, systems. Their basic idea is that every adaptive advance sets the stage (the context) for novelty at the next higher level, while this context also stabilizes the lower levels.

## References

[B1] Ahl V. Allen T. F. Allen T. F. H. (1996). *Hierarchy Theory: A Vision, Vocabulary, and Epistemology.* New York, NY: Columbia University Press.

[B2] Aleman B. Merker B. (2014). Consciousness without cortex: a hydranencephaly family survey. *Acta Paediatr.* 103 1057–1065. 10.1111/apa.12718 24942496

[B3] Allen T. F. Starr T. B. (1982). *Hierarchy: Perspectives for Ecological Complexity.* Chicago, IL: University of Chicago.

[B4] Andersen P. B. Emmeche C. Finnemann N. O. Christiansen P. V. (2000). *Downward Causation*: *Minds, Bodies and Matter*. Aarhus: Aarhus University Press.

[B5] Arendt D. Bertucci P. Y. Achim K. Musser J. M. (2019). Evolution of neuronal types and families. *Curr. Opin. Neurobiol.* 56 144–152. 10.1016/j.conb.2019.01.022 30826503 PMC6556553

[B6] Atmanspacher H. (2012). Identifying mental states from neural states under mental constraints. *Interface Focus* 2 74–81. 10.1098/rsfs.2011.0058 23386962 PMC3262303

[B7] Atmanspacher H. (2015). Contextual emergence of mental states. *Cogn. Process.* 16 359–364. 10.1007/s10339-015-0658-0 26018611

[B8] Atmanspacher H. beim Graben P. (2009). Contextual emergence. *Scholarpedia* 4:7997 10.4249/scholarpedia.7997 19731148

[B9] Beckermann A. Flohr H. Kim J. (eds) (2011). *Emergence or Reduction? Essays on the Prospects of Nonreductive Physicalism.* New York, NY: Walter de Gruyter.

[B10] Bedau M. A. (1997). Weak emergence. *Philos. Perspect.* 11 375–399. 10.1111/0029-4624.31.s11.17

[B11] Bedau M. A. (2008). “Downward causation and the autonomy of weak emergence,” in *Emergence: Contemporary Readings in Philosophy and Science*, eds Bedau M. A. Humphreys P. (Cambridge, MA: MIT Press), 155–188.

[B12] Bedau M. A. Humphreys P. (2008). *Emergence: Contemporary Readings in Philosophy and Science.* Cambridge, MA: MIT Press.

[B13] beim Graben P. (2014). Contextual emergence of intentionality. *J. Conscious. Stud.* 21 75–96. 10.1007/s12124-010-9117-8 20349217

[B14] Berridge K. C. Kringelbach M. L. (2015). Pleasure systems in the brain. *Neuron* 86 646–664. 10.1016/j.neuron.2015.02.018 25950633 PMC4425246

[B15] Bickle J. (2019). *Multiple Realizability. Encyclopedia of Cognitive Science.* Available online at: https://plato.stanford.edu/archives/spr2019/entries/multiple-realizability (accessed May 14, 2020).

[B16] Bishop R. C. Atmanspacher H. (2006). Contextual emergence in the description of properties. *Found. Phys.* 36 1753–1777. 10.1007/s10701-006-9082-8

[B17] Block N. (1980). Troubles with functionalism. *Read. Philos. Psychol.* 1 268–305.

[B18] Block N. (1995). On a confusion about a function of consciousness. *Behav. Brain Sci.* 18 227–247. 10.1017/S0140525X00038188

[B19] Block N. (2012). The grain of vision and the grain of attention. *Thought J. Philos.* 1 170–184. 10.1002/tht3.28

[B20] Bosch T. C. Klimovich A. Domazet-Lošo T. Gründer S. Holstein T. W. Jékely G. (2017). Back to the basics: cnidarians start to fire. *Trends Neurosci.* 40 92–105. 10.1016/j.tins.2016.11.005 28041633 PMC5285349

[B21] Broad C. D. (1925). *The Mind and its Place in Nature.* London: Routledge.

[B22] Bronfman Z. Z. Ginsburg S. Jablonka E. (2016). The transition to minimal consciousness through the evolution of associative learning. *Front. Psychol.* 7:1954. 10.3389/fpsyg.2016.01954 28066282 PMC5177968

[B23] Brunet T. Larson B. T. Linden T. A. Vermeij M. J. McDonald K. King N. (2019). Light-regulated collective contractility in a multicellular choanoflagellate. *Science* 366 326–344. 10.1101/661009 31624206

[B24] Brunk C. F. Martin W. F. (2019). Archaeal histone contributions to the origin of eukaryotes. *Trends Microbiol.* 27 703–714. 10.1016/j.tim.2019.04.002 31076245

[B25] Cabanac M. (1996). On the origin of consciousness, a postulate and its corollary. *Neurosci. Biobehav. Rev.* 20 33–40. 10.1016/0149-7634(95)00032-A 8622827

[B26] Carr J. (1981). *Applications of Centre Manifold Theory.* Berlin: Springer-Verlag.

[B27] Carruthers P. (2016). *Higher-Order Theories of Consciousness. Stanford Encyclopedia of Philosophy.* Available online at: https://plato.stanford.edu/archives/fall2016/entries/consciousness-higher/ (accessed May 14, 2020).

[B28] Chalmers D. J. (1995). Facing up to the problem of consciousness. *J. Conscious. Stud.* 2 200–219.

[B29] Chalmers D. J. (1996). *The Conscious Mind: In Search of a Fundamental Theory.* New York, NY: Oxford University Press.

[B30] Chalmers D. J. (2006). “Strong and weak emergence,” in *The Re-Emergence of Emergence: The Emergentist Hypothesis from Science to Religion*, eds Clayton P. Davies P. (Oxford: Oxford University Press), 244–254.

[B31] Chica A. B. Lasaponara S. Lupiáñez J. Doricchi F. Bartolomeo P. (2010). Exogenous attention can capture perceptual consciousness: ERP and behavioural evidence. *Neuroimage* 51 1205–1212. 10.1016/j.neuroimage.2010.03.002 20211272

[B32] Choifer A. (2018). A new understanding of the first-person and third-person perspectives. *Philos. Pap.* 47 333–371. 10.1080/05568641.2018.1450160 24187537

[B33] Churchland P. M. (2013). *Matter and Consciousness.* Cambridge, MA: MIT press.

[B34] Clark A. (2013). Whatever next? Predictive brains, situated agents, and the future of cognitive science. *Behav. Brain Sci.* 36 181–204. 10.1017/S0140525X12000477 23663408

[B35] Clayton P. (2006). “Conceptual foundations of emergence theory,” in *The Re-Emergence of Emergence: The Emergentist Hypothesis from Science to Religion*, eds Clayton P. Davies P. (Oxford: Oxford University Press), 1–31.

[B36] Clayton P. Davies P. (2006). *The Re-Emergence of Emergence: The Emergentist Hypothesis from Science To Religion.* Oxford: Oxford University Press.

[B37] Conee E. (1994). Phenomenal knowledge. *Aust. J. Philos.* 72 136–150. 10.1080/00048409412345971

[B38] Damasio A. (2010). *Self Comes to Mind: Constructing the Conscious Brain.* New York, NY: Vintage.

[B39] Davies P. (2006). “Preface,” in *The Re-Emergence of Emergence*: *The Emergentist Hypothesis from Science to Religion*, eds Clayton P. Davies P. (Oxford: Oxford University Press), ix–xiv.

[B40] Deacon T. W. (2011). *Incomplete Nature: How Mind Emerged from Matter.* New York, NY: WW Norton and Company.

[B41] Denton D. (2005). *The Primordial Emotions: The Dawning of Consciousness.* Oxford: Oxford University Press.

[B42] Edelman D. B. Baars B. J. Seth A. K. (2005). Identifying hallmarks of consciousness in non-mammalian species. *Conscious. Cogn.* 14 169–187. 10.1016/j.concog.2004.09.001 15766896

[B43] Ellis G. (2006). “On the nature of emergent reality,” in *The Re-Emergence of Emergence: The Emergentist Hypothesis from Science to Religion*, eds Clayton P. Davies P. (Oxford: Oxford University Press), 79–107.

[B44] Emmeche C. Køppe S. Stjernfelt F. (2000). “Levels, emergence, and three versions of downward causation,” in *Downward Causation. Minds, Bodies and Matter*, eds Andersen P. B. Emmeche C. Finnemann N. O. Christiansen P. V. (Århus: Aarhus University Press), 13–34.

[B45] Endicott R. P. (1993). Species-specific properties and more narrow reductive strategies. *Erkenntnis* 38 303–321.

[B46] England J. L. (2013). Statistical physics of self-replication. *J. Chem. Phys.* 139:121923 10.1063/1.4818538 24089735

[B47] Erwin D. H. Valentine J. W. (2013). *The Cambrian Explosion: The Construction of Animal Biodiversity.* Greenwood Village, CO: Roberts and Co.

[B48] Feigl H. (1958). *The ‘Mental’ and the ‘Physical’.* Minneapolis, MN: University of Minnesota Press.

[B49] Feinberg T. E. (2001). Why the mind is not a radically emergent feature of the brain. *J. Conscious. Stud.* 8 123–145.

[B50] Feinberg T. E. (2012). Neuroontology, neurobiological naturalism, and consciousness: a challenge to scientific reduction and a solution. *Phys. Life Rev.* 9 13–34. 10.1016/j.plrev.2011.10.019 22056393

[B51] Feinberg T. E. Mallatt J. (2013). The evolutionary and genetic origins of consciousness in the Cambrian Period over 500 million years ago. *Front. Psychol.* 4:667. 10.3389/fpsyg.2013.00667 24109460 PMC3790330

[B52] Feinberg T. E. Mallatt J. (2016a). *The Ancient Origins of Consciousness: How the Brain Created Experience.* Cambridge, MA: MIT Press.

[B53] Feinberg T. E. Mallatt J. (2016b). The nature of primary consciousness: a new synthesis. *Conscious. Cogn.* 43 113–127. 10.1016/j.concog.2016.05.009 27262691

[B54] Feinberg T. E. Mallatt J. (2018a). *Consciousness Demystified.* Cambridge, MA: MIT Press.

[B55] Feinberg T. E. Mallatt J. (2018b). *Unlocking the “Mystery” of Consciousness. Scientific American, Observations.* Available online at: https://blogs.scientificamerican.com/observations/unlocking-the-mystery-of-consciousness (accessed January 13, 2020).

[B56] Feinberg T. E. Mallatt J. M. (2019). Subjectivity “demystified”: neurobiology, evolution, and the explanatory gap. *Front. Psychol.* 10:1686. 10.3389/fpsyg.2019.01686 31417451 PMC6685416

[B57] Gershman S. J. Horvitz E. J. Tenenbaum J. B. (2015). Computational rationality: a converging paradigm for intelligence in brains, minds, and machines. *Science* 349 273–278. 10.1126/science.aac6076 26185246

[B58] Ginsburg S. Jablonka E. (2019). *The Evolution of the Sensitive Soul: Learning and the Origins of Consciousness.* Cambridge, MA: MIT Press.

[B59] Godfrey-Smith P. (2016). “Animal evolution and the origins of experience” in *How Biology Shapes Philosophy: New Foundations for Naturalism*, ed. Smith D. L. (Cambridge: Cambridge University Press), 23–50.

[B60] Godfrey-Smith P. (2019). *Evolving Across the Explanatory Gap. Philosophy, Theory, and Practice in Biology.* Available online at: https://quod.lib.umich.edu/cgi/t/text/text-idx?cc=ptpbio;c=ptb;c=ptpbio;idno=16039257.0011.001;g=ptpbiog;rgn=main;view=text;xc=1 (accessed October 11, 2018).

[B61] Goff P. Seager W. Allen-Hermanson S. (2017). *Panpsychism. The Stanford Encyclopedia of Philosophy.* Available online at: https://plato.stanford.edu/archives/win2017/entries/panpsychism/ (accessed May 14, 2020).

[B62] Grillner S. El Manira A. (2020). Current principles of motor control, with special reference to vertebrate locomotion. *Physiol. Rev.* 100 271–320. 10.1152/physrev.00015.2019 31512990

[B63] Haken H. (1983). *Synergetics: An Introduction. Non-Equilibrium Phase Transition and Self-Organization in Physics.* Berlin: Springer-Verlag.

[B64] Hasan A. Fumerton R. (2019). *Knowledge by Acquaintance vs. Description. The Stanford Encyclopedia of Philosophy.* Available online at: https://plato.stanford.edu/archives/sum2019/entries/knowledge-acquaindescrip/ (accessed May 14, 2020).

[B65] Hodge R. D. Bakken T. E. Miller J. A. Smith K. A. Barkan E. R. Graybuck L. T. (2019). Conserved cell types with divergent features in human versus mouse cortex. *Nature* 573 61–68. 10.1038/s41586-019-1506-7 31435019 PMC6919571

[B66] Hu H. (2016). Reward and aversion. *Annu. Rev. Neurosci.* 39 297–324. 10.1146/annurev-neuro-070815-014106 27145915

[B67] Jackson F. (1982). Epiphenomenal qualia. *Philos. Q.* 32 127–136. 10.2307/2960077 9854266

[B68] Jackson F. (1986). What Mary didn’t know. *J. Philos.* 83 291–295. 10.2307/2026143 16422045

[B69] Jordan J. S. Ghin M. (2006). (Proto-) consciousness as a contextually emergent property of self-sustaining systems. *Mind Matter* 4 45–68.

[B70] Jylkkä J. Railo H. (2019). Consciousness as a concrete physical phenomenon. *Conscious. Cogn.* 74 102779. 10.1016/j.concog.2019.102779 31295656

[B71] Kim J. (1992). Multiple realization and the metaphysics of reduction. *Philos. Phenomenol. Res.* 52 1–26.

[B72] Kim J. (1998). *Mind in a Physical World: An Essay on the Mind–Body Problem and Mental Causation.* Cambridge, MA: MIT Press.

[B73] Kim J. (2006). “Being realistic about emergence,” in *The Re-Emergence of Emergence* : *The Emergentist Hypothesis from Science to Religion*, eds Clayton P. Davies P. (Oxford: Oxford University Press), 190–202.

[B74] Klein C. Barron A. B. (2016). Insects have the capacity for subjective experience. *Anim. Sent.* 9 1–19. 10.1073/pnas.1520084113 27091981 PMC4983823

[B75] Koch C. (2019). *The Feeling of Life Itself.* Cambridge, MA: MIT Press.

[B76] Koch C. Massimini M. Boly M. Tononi G. (2016). Neural correlates of consciousness: progress and problems. *Nat. Rev. Neurosci.* 17 307–321. 10.1038/nrn.2016.22 27094080

[B77] Lacalli T. (2018). Amphioxus neurocircuits, enhanced arousal, and the origin of vertebrate consciousness. *Conscious. Cogn.* 62 127–134. 10.1016/j.concog.2018.03.006 29598920

[B78] Lamme V. A. (2006). Towards a true neural stance on consciousness. *Trends Cogn. Sci.* 23 571–579. 10.1016/j.tics.2006.09.001 16997611

[B79] Lane N. Martin W. (2010). The energetics of genome complexity. *Nature* 467 929. 10.1038/nature09486 20962839

[B80] Levine J. (1983). Materialism and phenomenal properties: the explanatory gap. *Pac. Philos. Q.* 64 354–361. 10.1111/j.1468-0114.1983.tb00207.x 23073546

[B81] Lewes G. H. (1877). *Problems of Life and Mind.* London: Trübner & Company.

[B82] Mallatt J. Feinberg T. E. (2017). Consciousness is not inherent in but emergent from life. *Anim. Sent.* 1 1–7.

[B83] Mayr E. (1982). *The Growth of Biological Thought: Diversity, Evolution, and Inheritance.* Cambridge, MA: Harvard University Press.

[B84] Mayr E. (2004). *What Makes Biology Unique? Considerations on the Autonomy of a Scientific Discsipline.* Cambridge: Cambridge University Press.

[B85] Merker B. (2007). Consciousness without a cerebral cortex: a challenge for neuroscience and medicine. *Behav. Brain Sci.* 30 63–81. 10.1017/S0140525X07000891 17475053

[B86] Metzinger T. (2003). *Being No One: The Self-Model Theory of Subjectivity.* Cambridge, MA: MIT Press.

[B87] Metzinger T. (ed.) (1995). *Conscious Experience.* Thorverton: Imprint Academic.

[B88] Mogensen J. Overgaard M. (2017). Reorganization of the connectivity between elementary functions–A model relating conscious states to neural connections. *Front. Psychol.* 8:625. 10.3389/fpsyg.2017.00625 28473797 PMC5397468

[B89] Morowitz H. J. (2002). *The Emergence of Everything: How the World Became Complex.* New York, NY: Oxford University Press.

[B90] Nagel T. (1974). What is it like to be a bat? *Philos. Rev.* 83 435–450.

[B91] Nagel T. (1986). *The View from Nowhere.* New York, NY: Oxford University press.

[B92] Natarajan C. Hoffmann F. G. Weber R. E. Fago A. Witt C. C. Storz J. F. (2016). Predictable convergence in hemoglobin function has unpredictable molecular underpinnings. *Science* 354 336–339. 10.1126/science.aaf9070 27846568 PMC5464326

[B93] Nida-Rümelin M. O Conaill D. (2019). *Qualia: The Knowledge Argument. The Stanford Encyclopedia of Philosophy.* Available online at: https://plato.stanford.edu/archives/win2019/entries/qualia-knowledge/ (accessed May 14, 2020).

[B94] Noble R. Tasaki K. Noble P. J. Noble D. (2019). Biological relativity requires circular causality but not symmetry of causation: so, where, what and when are the boundaries? *Front. Physiol.* 10:827. 10.3389/fphys.2019.00827 31379589 PMC6656930

[B95] Northcutt R. G. (2012). Evolution of centralized nervous systems: two schools of evolutionary thought. *Proc. Natl. Acad. Sci. U.S.A.* 109 (Suppl. 1), 10626–10633. 10.1073/pnas.1201889109 22723354 PMC3386872

[B96] Nunez P. L. (2016). *The New Science of Consciousness.* Amherst, NY: Prometheus Books.

[B97] Panksepp J. (2004). *Affective Neuroscience: The Foundations of Human and Animal Emotions.* New York, NY: Oxford University Press.

[B98] Panksepp J. (2016). The cross-mammalian neurophenomenology of primal emotional affects: from animal feelings to human therapeutics. *J. Comp. Neurol.* 524 1624–1635. 10.1002/cne.23969 26876723

[B99] Pattee H. H. (1970). “The problem of biological hierarchy,” in *Towards a Theoretical Biology 3, Drafts*, ed. Waddington C. H. (Edinburgh: Edinburgh University Press), 117–136.

[B100] Plotnick R. E. Dornbos S. Q. Chen J. (2010). Information landscapes and sensory ecology of the Cambrian Radiation. *Paleobiology* 36 303–317. 10.1666/08062.1

[B101] Popper K. R. Eccles J. C. (1977). *The Self and its Brain.* New York, NY: Springer.

[B102] Putnam H. (1967). “Psychological predicates,” in *Art, Mind, and Religion*, eds Capitan W. H. Merrill D. D. (Pittsburgh, PA: University of Pittsburgh Press), 37–48.

[B103] Revonsuo A. (2006). *Inner Presence: Consciousness as a Biological Phenomenon.* Cambridge, MA: MIT Press.

[B104] Revonsuo A. (2010). *Consciousness: The Science of Subjectivity.* Hove: Psychology Press.

[B105] Rothschild L. J. (2006). “The role of emergence in biology,” in *The Re-Emergence of Emergence* : *The Emergentist Hypothesis from Science to Religion*, eds Clayton P. Davies P. (Oxford: Oxford University Press), 151–165.

[B106] Russell B. (1910). Knowledge by acquaintance and knowledge by description”. *Proc. Arist. Soc.* 11 108–128.

[B107] Russell B. (1912). *The Problems of Philosophy.* New York, NY: Henry Holt and Company.

[B108] Russell B. (1914). On the nature of acquaintance. *Monist* 24 161–187.

[B109] Salthe S. N. (1985). *Evolving Hierarchical Systems: Their Structure and Representation.* New York, NY: Columbia University Press.

[B110] Scott A. (1995). *Stairway to the Mind: The Controversial New Science of Consciousness.* New York, NY: Springer-Verlag.

[B111] Searle J. R. (1992). *The Rediscovery of the Mind.* Cambridge, MA: MIT Press.

[B112] Searle J. R. (2007). Dualism revisited. *J. Physiol. Paris* 101 169–178. 10.1016/j.jphysparis.2007.11.003 18276124

[B113] Seth A. (2009). Explanatory correlates of consciousness: theoretical and computational challenges. *Cogn. Comput.* 1 50–63. 10.1007/s12559-009-9007-x

[B114] Shear J. (ed.) (1999). *Explaining Consciousness: The Hard Problem.* Cambridge, MA: MIT Press.

[B115] Shigeno S. Murakami Y. Nomura T. (eds) (2017). *Brain Evolution by Design: From Neural Origin to Cognitive Architecture.* New York, NY: Springer.

[B116] Siciliano C. A. Noamany H. Chang C. J. Brown A. R. Chen X. Leible D. (2019). A cortical-brainstem circuit predicts and governs compulsive alcohol drinking. *Science* 366 1008–1012. 10.1126/science.aay1186 31754002 PMC6989100

[B117] Simon H. (1973). “The organization of complex systems,” in *Hierarchy Theory: The Challenge of Complex Systems*, ed. Patee H. H. (New York, NY: George Braziller), 1–27.

[B118] Skrbina D. (ed.) (2009). *Mind that Abides: Panpsychism in the New Millennium*, Vol. 75 Amsterdam: John Benjamins Publishing.

[B119] Solms M. (2019). The hard problem of consciousness and the free energy principle. *Front. Psychol.* 9:2714. 10.3389/fpsyg.2018.02714 30761057 PMC6363942

[B120] Sperry R. W. (1984). Consciousness, personal identity and the divided brain. *Neuropsychologia* 22 661–673. 10.1016/0028-3932(84)90093-9 6084824

[B121] Sperry R. W. (1990). “Forebrain commissurotomy and conscious awareness,” in *Brain Circuits and Functions of the Mind*, ed. Trevarthen C. (New York, NY: Cambridge University Press), 371–388.

[B122] Stayton C. T. (2015). What does convergent evolution mean? The interpretation of convergence and its implications in the search for limits to evolution. *Interface Focus* 5:20150039. 10.1098/rsfs.2015.0039 26640646 PMC4633856

[B123] Strausfeld N. J. (2012). *Arthropod Brains: Evolution, Functional Elegance, and Historical Significance.* Cambridge, MA: Belknap Press of Harvard University Press.

[B124] Szõnyi A. Zichó K. Barth A. M. Gönczi R. T. Schlingloff D. Török B. (2019). Median raphe controls acquisition of negative experience in the mouse. *Science* 366:eaay1094. 10.1126/science.aay8746 31780530

[B125] Taiz L. Alkon D. Draguhn A. Murphy A. Blatt M. Thiel G. (2020). Reply to Trewavas et al. and Calvo and Trewavas. *Trends Plant Sci.* 25 218–220. 10.1016/j.tplants.2019.12.020 31926764

[B126] Teller P. (2011). “Subjectivity and knowing what it’s like,” in *Emergence or Reduction? Essays on the Prospects of Nonreductive Physicalism*, eds Beckermann A. Flohr H. Kim J. (New York, NY: Walter de Gruyter), 180–200.

[B127] Thompson E. (2007). *Mind in Life: Biology, Phenomenology and the Sciences of Mind.* Cambridge, MA: Harvard University Press.

[B128] Tononi G. Koch C. (2015). Consciousness: here, there and everywhere? *Philos. Trans. R. Soc. B Biol. Sci.* 370:20140167. 10.1098/rstb.2014.0167 25823865 PMC4387509

[B129] Trestman M. (2013). The Cambrian explosion and the origins of embodied cognition. *Biol. Theory* 8 80–92. 10.1007/s13752-013-0102-6

[B130] Tschacher W. Haken H. (2007). Intentionality in non-equilibrium systems? The functional aspects of self-organized pattern formation. *New Ideas Psychol.* 25 1–15. 10.1016/j.newideapsych.2006.09.002

[B131] Tsuchiya N. Koch C. (2008). “The relationship between consciousness and attention,” in *The Neurology of Consciousness: Cognitive Neuroscience and Neuropathology*, eds Tononi G. Laureys S. (Oxford: Elsevier Academic), 63–78.

[B132] Tsuchiya N. Van Boxtel J. J. (2013). Introduction to research topic: attention and consciousness in different senses. *Front. Psychol.* 4:249. 10.3389/fpsyg.2013.00249 23641230 PMC3640185

[B133] Tye M. (2002). *Consciousness, Color, and Content.* Cambridge, MA: MIT Press.

[B134] Van Gulick R. (2001). Reduction, emergence and other recent options on the mind/body problem. A philosophic overview. *J. Conscious. Stud.* 8 1–34.

[B135] Van Kranendonk M. J. Deamer D. W. Djokic T. (2017). Life springs. *Sci. Am.* 317 28–35. 10.1038/scientificamerican0817-28 29565926

[B136] Velmans M. (1991). Consciousness from a first-person perspective. *Behav. Brain Sci.* 14 702–719.

[B137] Velmans M. (2008). Reflexive monism. *J. Conscious. Stud.* 15 5–50.

[B138] Watson T. (2019). The trickster microbes shaking up the tree of life. *Nature* 569 323–324. 10.1038/d41586-019-01496-w 31089235

[B139] Witherington D. C. (2011). Taking emergence seriously: the centrality of circular causality for dynamic systems approaches to development. *Hum. Dev.* 54 66–92. 10.1159/000326814

